# PyCoMo: a python package for community metabolic model creation and analysis

**DOI:** 10.1093/bioinformatics/btae153

**Published:** 2024-03-26

**Authors:** Michael Predl, Marianne Mießkes, Thomas Rattei, Jürgen Zanghellini

**Affiliations:** Department of Microbiology and Ecosystem Science, Division of Computational Systems Biology, Centre for Microbiology and Environmental Systems Science, University of Vienna, 1030 Vienna, Austria; Doctoral School in Microbiology and Environmental Science, University of Vienna, 1030 Vienna, Austria; Department of Analytical Chemistry, Faculty of Chemistry, University of Vienna, 1090 Vienna, Austria; Austrian Centre of Industrial Biotechnology, 1190 Vienna, Austria; Department of Microbiology and Ecosystem Science, Division of Computational Systems Biology, Centre for Microbiology and Environmental Systems Science, University of Vienna, 1030 Vienna, Austria; Doctoral School in Microbiology and Environmental Science, University of Vienna, 1030 Vienna, Austria; Department of Analytical Chemistry, Faculty of Chemistry, University of Vienna, 1090 Vienna, Austria; Austrian Centre of Industrial Biotechnology, 1190 Vienna, Austria

## Abstract

**Summary:**

PyCoMo is a python package for quick and easy generation of genome-scale compartmentalized community metabolic models that are compliant with current openCOBRA file formats. The resulting models can be used to predict (i) the maximum growth rate at a given abundance profile, (ii) the feasible community compositions at a given growth rate, and (iii) all exchange metabolites and cross-feeding interactions in a community metabolic model independent of the abundance profile; we demonstrate PyCoMo’s capability by analysing methane production in a previously published simplified biogas community metabolic model.

**Availability and implementation:**

PyCoMo is freely available under an MIT licence at http://github.com/univieCUBE/PyCoMo, the Python Package Index, and Zenodo.

## 1 Introduction

Cross-feeding interactions are crucial for community formation and function. Predicting these interactions is an open challenge, with much development in the field of metabolic modelling (Heinken *et al.* 2021). It has expanded in the direction of microbial communities, with research being done on simple, cultured communities up to personalized models of the human gut microbiome (Diener *et al.* 2020, Ibrahim *et al.* 2021, Saa *et al.* 2022).

Metabolic modelling on the level of communities allows investigating questions that can only be stated in the context of communities, such as which interactions can occur within them. Different approaches have been developed to model communities of metabolic models: individual modelling of the members with iterative cross-talk via metabolite availability, lumped models representing the collective enzymatic capabilities of a community without cellular borders, and compartmentalized metabolic models, which place each member model in a separate compartment and add a shared exchange compartment for interaction (Perez-Garcia *et al.* 2016). While resulting in the largest and most complex models, the compartmentalized approach allows direct modelling of inter-species interactions such as cross-feeding.

The creation of compartmentalized community metabolic models includes many technical obstacles, such as matching the namespace of exchange metabolites, creating a community biomass function and preserving the attribution of metabolites and reactions to the community members within the model. All these tasks can be automated when all necessary information is present, as shown in Commmodelpy by Bekiaris and Klamt (2021). Yet, the models generated by the current version (0.0.3) of Commmodelpy face several issues that hinder their reusability for further analyses and curation: the information which member a gene or compartment belongs to is not automatically retained, the flux bounds are changed due to normalization, and the abundance composition cannot be changed without rerunning the merging procedure.

While standard formats for single organism metabolic models like SBML (Keating *et al.* 2020) have emerged and been widely adopted, no standard format for community metabolic models has been established yet. This not only poses a challenge for the compatibility and reuse of community metabolic models, but can also lead to a loss of information in the community model representation, as is the case for Commmodelpy. Apart from retaining information, the abundances of community members and any changes to the original flux bounds need to be documented as well for the model to be reusable.

Here, we present PyCoMo, a python package for automated generation of compartmentalized community metabolic models. The generated models are suited for reuse and continuous improvement, as all information of the single organisms, as well as all community information, is stored in a format complying to existing standards. PyCoMo allows the quick calculation of the set of potential metabolite exchanges and resulting interactions across the entire community composition space, with a single flux variability analysis (FVA) run. We show an example of these functionalities by replicating the analysis of a previously published three species biogas community (Koch *et al.* 2019).

## 2 Description

**Figure 1.**
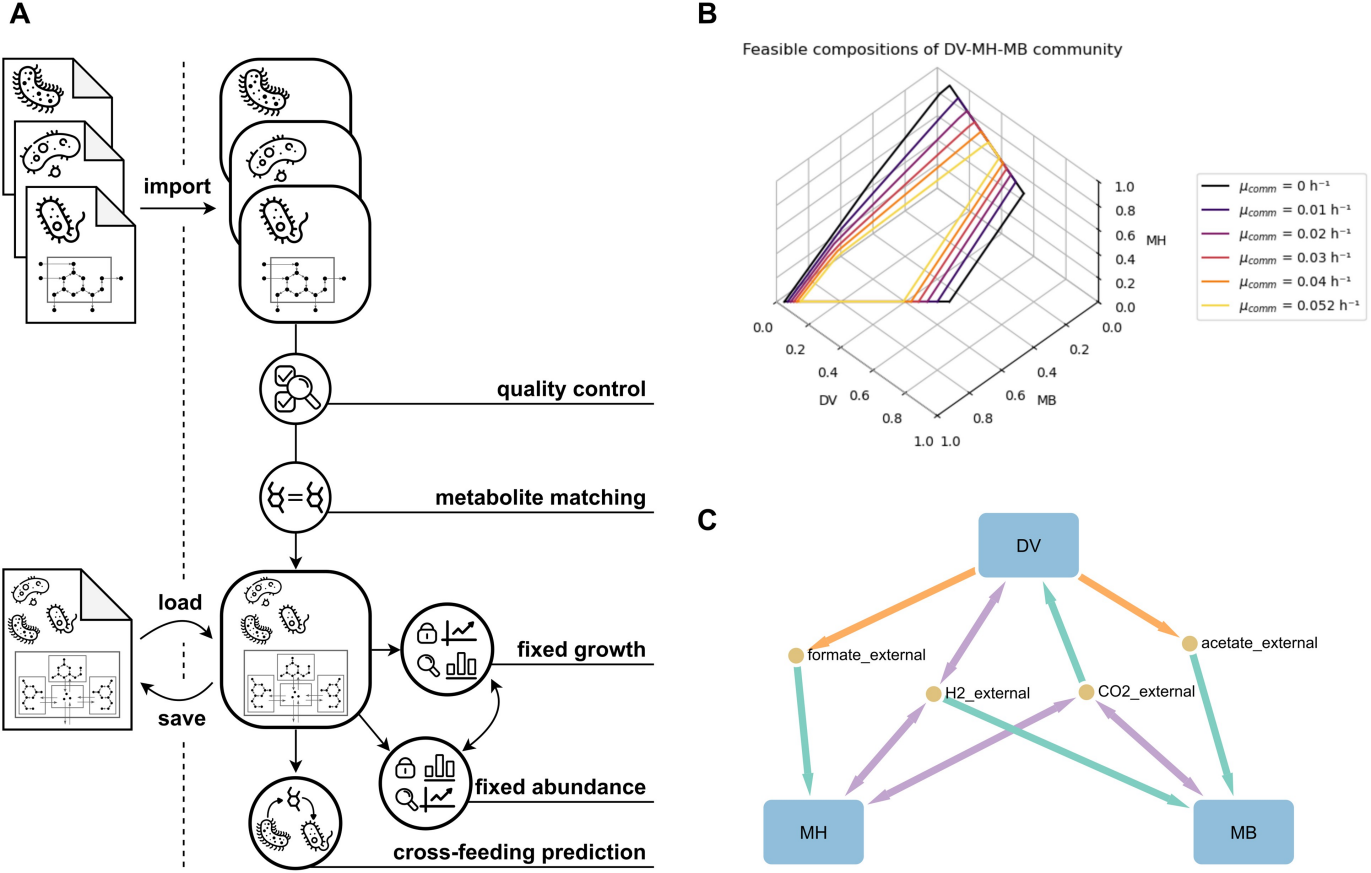
Summary of PyCoMo. (A) Overview of the automated steps in compartmentalized community metabolic model creation and the available analysis. All components of the final model can be traced back to their community member of origin. The community metabolic models can be saved and loaded into SBML format while retaining the original flux bounds, making the models reusable. Mass and charge balance of the entire model is checked, including all external metabolites matched between community members. The community metabolic model structure can be set to fixed growth or fixed abundance. All functionalities are available via a command-line interface. (B) Feasible compositions at different community growth rates in a three species biogas community (Koch *et al.* 2019). DV, *Desulfovibrio vulgaris*; MH, *Methanospirillum hungatei*; MB, *Methanosarcina barkeri*. The medium is specified to contain ethanol and CO_2_, with ethanol being the substrate for *Desulfovibrio vulgaris*, which produces the substrates for the other community members. (C) Visualization of the community metabolic model and its cross-feeding interactions using ScyNet. The metabolite exchanges were calculated independently of the community composition and growth rate. Arrow colours denote feasible metabolite production (orange), uptake (green) or both (violet) by the communities’ members.

PyCoMo is implemented in Python 3 and utilizes COBRApy (Ebrahim *et al.* 2013) for model input and output, as well as for fundamental structure and analysis methods. Three types of functionality for community metabolic modelling are implemented: (i) the preparation of the single organism models before merging into a community metabolic model, (ii) the generation of the community metabolic model in a structured format, and (iii) the contextualization and analysis of the community metabolic model (Fig. 1A). The resulting community model can then be used to predict the maximal community growth rate (at a given composition) or all feasible community compositions (at a given community growth rate).

The input for PyCoMo are the metabolic models of the community members, in any of the file formats supported by COBRApy (SBML, MATLAB, JSON, and YAML). The imported member models are prepared for merging, by prefixing all individual organism objects’ (i.e. gene products, species, reactions, etc.) IDs with the name of the member they belong to. Further, a separate “medium compartment” is added, which will allow metabolite exchange between organisms as well as the environment. Existing boundary reactions are converted into (internal) reactions transporting metabolites from the organisms’ compartments to the medium compartment, and new boundary reactions are set for the medium compartment. Additionally, consistency checks and corrections for compliance with the SBML format (Keating *et al.* 2020) are performed.

Boundary metabolites of all members need to be matched in the medium compartment. PyCoMo resolves the matching either by ID, or by annotation. When matching by name, all metabolites with the same SBML ID are matched. Matching by annotation utilizes the information of database cross-referencing in the metabolite annotations. Such annotations are available to varying extent in all automatically generated metabolic models. The user can select a database to match all metabolites in the medium compartment with a reference to the same metabolite in the specified database. To avoid inconsistent matches, this process will only be followed through if all boundary metabolites map uniquely to the annotation database, otherwise PyCoMo stops and informs the user to use a different database or match the metabolites via names.

In a second step a community metabolic model object is created, using the prepared models as input. Here, all components of the models are merged. Metabolites, reactions, compartments and genes remain affiliated to their origin, due to the name change in the preparation.

Solving a community metabolic model leads to a nonlinear problem by default, due to the bilinear dependence of the community growth rate on the community abundance profile, and the growth rate of the individual members (Koch *et al.* 2019). To linearize the model, one can either hold the growth rate or the community member fraction constant by constraining the corresponding reactions (see Supplementary Material).

Typically, the fluxes in metabolic models are normalized to the (dry) mass of the system. In the context of microbial communities, this system mass represents the total dry mass of the community. Consequently, when consolidating multiple models, all cell-specific fluxes, including their corresponding bounds, need to be re-scaled to match the total community mass. This can be efficiently achieved by multiplying the cell-specific fluxes (alongside their bounds) by the mass fraction of the specific member in the community (Koch *et al.* 2019). However, when dealing with variable abundance profiles, flux bounds become dynamic, a characteristic not accommodated by the current SBML standard (level 3, Flux Balance Constraints Version 2 Release 2) (Olivier and Bergmann 2018). To address this, we convert each genome-scale metabolic model into an equivalent bound-free form, where flux bounds are represented as stoichiometric coefficients for newly introduced dummy metabolites. These metabolites are both produced and consumed by additional reactions, with their fluxes corresponding to the respective member’s mass fraction within the community (refer to Supplementary Material for details). This approach aligns with current standards, ensuring seamless interchangeability among various openCOBRA tools.

The result of the merging and these additional steps is always a COBRApy model object, allowing the large environment of methods available to be utilized. On top of the COBRApy analysis methods, PyCoMo includes the functionality to calculate cross-feeding interactions in the community metabolic model. As community metabolic models have the additional parameter of community composition, typical steady-state, constraint-based modelling either only represents a single state of the community, or is computationally intensive, as it needs to account for the whole space of abundance combinations. By setting the model structure to variable community composition, but also removing the constraint of equal growth rate among the community members, FVA of the members’ exchange reactions calculates the possible ranges of all metabolite exchanges and cross-feeding interactions. The resulting flux ranges may include cross-feeding interactions not feasible under balanced (community) growth if the production of metabolites is coupled to biomass production.

Community metabolic models generated by PyCoMo can easily be exported and transferred, as all the included export functionalities of COBRApy can be applied, and SBML format requirements are checked. PyCoMo was tested to work with input models of different origins [AGORA (Heinken *et al.* 2023), BIGG (King *et al.* 2016), CarveMe (Machado *et al.* 2018), PathwayTools (Karp *et al.* 2021), gapseq (Zimmermann *et al.* 2021)], although any model that can be imported using COBRApy will work. Further, community metabolic models generated by PyCoMo can be easily visualized using ScyNet (github.com/univieCUBE/ScyNet, manuscript submitted; see Fig. 1C), as all format requirements are fulfilled.

PyCoMo contains a command line interface, allowing to directly generate community metabolic models, reading all member models from file and writing the resulting community metabolic model to file. All functionalities described above are available within the command line interface and thus facilitate an easy integration into existing workflows.

## 3 Results

As an example of a possible workflow, we have analysed a simplified biogas community of three species, aiming to construct a community metabolic model and compare all potential cross-feeding interactions to literature knowledge (Koch *et al.* 2019). The community consists of *Desulfovibrio vulgaris*, *Methanospirillum hungatei* and *Methanosarcina barkeri*, each representing a functional guild in the metabolic model. The members of the community are known to be actively cross-feeding, producing methane from ethanol or lactate, via the intermediary metabolites acetate, formate, H_2_ and CO_2_ (Koch *et al.* 2016). The models of the species members have been previously generated by Koch *et al.* (2019).

A community metabolic model is generated with PyCoMo following the workflow depicted in Fig. 1A. The input models are loaded from SBML files and the exchange metabolites are matched via identical IDs. The medium is set to contain ethanol and CO_2_, as specified by Koch *et al.* (2019). The potential exchange metabolites and cross-feeding interactions were calculated. The result (Fig. 1B) shows that cross-feeding can occur via acetate, H_2_, CO_2_, and formate. For a clear representation of the interaction structure in the community, the community metabolic model and its cross-feeding interactions is visualized with ScyNet (Fig. 1C).

FVA of the generated community model shows that *D.vulgaris* can provide acetate and hydrogen to *M.barkeri*. These metabolites are known substrates for two different pathways for methanogenesis in *M.barkeri*, the acetoclastic pathway and the hydrogenotrophic pathway. Similarly, *D.vulgaris* can provide formate and hydrogen for *M.hungatei*, which are all processed via the hydrogenotrophic pathway.

The community metabolic model would also allow *D.vulgaris* to take up H_2_ and CO_2_. This uptake was not found in the analysis by Koch *et al.* (2019). While it is known that *D.vulgaris* can grow on acetate and hydrogen with sulphate as an electron acceptor, sulphate is not part of the growth medium, neither is acetate. PyCoMo could detect a thermodynamically infeasible cycle as the reason, allowing *D.vulgaris* to take up H_2_ (see Supplementary Material, Section 3). In this cycle, *D.vulgaris* produces formate from H_2_ and CO_2_. Subsequently, *M.hungatei* converts the formate back into H_2_ and CO_2_, without the need for any other metabolites.

The performance of PyCoMo was tested using communities of different sizes (5–40 members). Genome-scale metabolic models from the AGORA collection were used as input models. Three tasks were timed on a system with 16 GB RAM and an AMD Ryzen 7 5700G using only a single core: the construction of the community metabolic model, flux balance analysis (FBA) at equal community member abundance, and the calculation of all potential exchange metabolites. The runtime scales approximately linear with community size for the construction of the community metabolic model and FBA, while the runtime of the calculation of all potential exchange metabolites scales quadratic, with a runtime of 11.3, 2.3, and 31.5 min respectively, at a community size of 40 members (average over three repetitions, see Supplementary Material, Section 5).

## 4 Conclusion

PyCoMo simplifies the generation and analysis of community metabolic models. This allows quickly addressing questions of which metabolites might be consumed, produced or exchanged in a community of interest, harnessing the knowledge base aspect of metabolic models on a community level. PyCoMo manages the construction of community models within a matter of seconds to minutes, yet the limitations of steady-state metabolic modelling methods apply. This means that simple optimization techniques like FBA will be feasible for larger communities, while the runtime for flux range and sampling approaches like FVA grows faster and can only be applied to smaller communities. Still, PyCoMo can find all exchange metabolites and cross-feeding interactions via FVA in a matter of 3 min for a 10-member community using genome-scale metabolic models.

Assembling community metabolic models with PyCoMo is reproducible and the resulting metabolic models can easily be used for further analysis with other metabolic modelling methods, including objectives other than biomass and objective free methods. All current openCOBRA file formats are supported for model input and export, with strict adherence to the SBML format. PyCoMo even facilitates (manual) curation on the level of communities by creating community metabolic models that fully preserve all available annotation and documentation in an interoperable, reusable manner adaptable to the necessary structures for different linearization approaches.

Finally, due to its command-line interface, PyCoMo can be easily integrated into (existing) genome analysis and phenotyping pipelines.

## Supplementary Material

btae153_Supplementary_Data

## Data Availability

The data underlying this article are available in this article and its supplementary material, and at https://pypi.org/project/pycomo/.
