# Supplementary material for: PyCoMo: a python package for community metabolic model creation and analysis
Source: Bioinformatics. 2024 Mar 26;40(4):btae153. doi: 10.1093/bioinformatics/btae153 (PMC10990682; doi:10.1093/bioinformatics/btae153)
Supplement: btae153_Supplementary_Data [file btae153_supplementary_data.pdf]

Supplementary material

# PyCoMo: a python package for community metabolic model creation and analysis

**Michael Predl**,[1,2] **Marianne Mießkes**,[3,4] **Thomas Rattei**[1,2,*] **and Jürgen Zanghellini**[3,4,*]

[1]Division of Computational Systems Biology, Centre for Microbiology and Environmental Systems Science, University of Vienna, Vienna, Austria, [2]Doctoral School in Microbiology and Environmental Science, University of Vienna, Vienna, Austria, [3]Department of Analytical Chemistry, Faculty of Chemistry, University of Vienna, Vienna, Austria and [4]Austrian Centre of Industrial Biotechnology, Vienna, Austria

*To whom correspondence should be addressed.

### Availability and Implementation:

PyCoMo is freely available under an MIT licence at `http://github.com/univieCUBE/PyCoMo`, the Python Package Index and Zenodo (`https://doi.org/10.5281/zenodo.10431322`).

**Contact:** thomas.rattei@univie.ac.at, juergen.zanghellini@univie.ac.at

## 1. Equivalent, bound-free metabolic network

Consider a metabolic network represented by its stoichiometric matrix, $\boldsymbol{N} \in \mathbb{R}^{m \times r}$ containing the net stoichiometric coefficients of $m$ internal metabolites in $r$ reactions. The vector $\boldsymbol{r} \in \mathbb{R}^r$ denotes a flux distribution (flux vector) through the network, and its components $r_i$ with $i \in \{1, ..., r\}$ are the respective reaction rates or fluxes.

In steady state, any feasible flux distribution through the network is a solution of the flux polyhedron given by

$$\boldsymbol{N}\boldsymbol{r} = \boldsymbol{0}, \tag{1a}$$

$$\boldsymbol{l} \leq \boldsymbol{r} \leq \boldsymbol{u}, \tag{1b}$$

where the capacity constraints $\boldsymbol{l}$ and $\boldsymbol{u}$ denote the vectors of lower and upper bounds on the flux distribution. These limits also include irreversibility constraints, where $l_i = 0$ for irreversible reactions $i \in I_{\text{irr}}$.

By introducing the slack variable $f$ and non-negative slack variable vectors $\boldsymbol{s}^l \in \mathbb{R}^r_{\geq 0}$ and $\boldsymbol{s}^u \in \mathbb{R}^r_{\geq 0}$ (associated with the lower and upper bounds of the reactions, respectively), the inhomogeneous set of equations in (1) can be transformed into a (higher-dimensional) set of homogeneous equations (Klamt et al., 2017)

$$\begin{pmatrix} \boldsymbol{0} & \boldsymbol{N} & \boldsymbol{0} & \boldsymbol{0} \\ -\boldsymbol{l} & \boldsymbol{I}_r & -\boldsymbol{I}_r & \boldsymbol{0} \\ \boldsymbol{u} & -\boldsymbol{I}_r & \boldsymbol{0} & -\boldsymbol{I}_r \end{pmatrix} \begin{pmatrix} f \\ \boldsymbol{r} \\ \boldsymbol{s}^l \\ \boldsymbol{s}^u \end{pmatrix} = \boldsymbol{0}, \tag{2a}$$

$$\boldsymbol{s}^l \geq \boldsymbol{0}, \tag{2b}$$

$$\boldsymbol{s}^u \geq \boldsymbol{0}. \tag{2c}$$

Here $\boldsymbol{I}_r$ denotes the identity matrix of size $r$.

By introducing the normalization condition

$$f = 1, \tag{3}$$

the solution spaces for $\boldsymbol{r}$ in (1) and (2) become identical. Thus, (2) can be interpreted as an equivalent, yet "bound-free" metabolic network, represented by the (transformed) stoichiometric matrix

$$\tilde{\boldsymbol{N}} = \begin{pmatrix} 0 & \boldsymbol{N} & \boldsymbol{0} & \boldsymbol{0} \\ -\boldsymbol{l} & \boldsymbol{I}_r & -\boldsymbol{I}_r & \boldsymbol{0} \\ \boldsymbol{u} & -\boldsymbol{I}_r & \boldsymbol{0} & -\boldsymbol{I}_r \end{pmatrix},$$

and (transformed) flux vector

$$\tilde{\boldsymbol{r}} = \begin{pmatrix} f & \boldsymbol{r} & \boldsymbol{s}^l & \boldsymbol{s}^u \end{pmatrix}^{\mathrm{T}},$$

including the irreversible reactions $\boldsymbol{s}^l$, and $\boldsymbol{s}^u$. Note that for bound-free models of the community members the normalization

condition $f$ introduced in equation 3 can also serve as a scaling factor, which enables one to rescale reaction fluxes and their bounds. This will prove useful in the context of the community metabolic models, where reaction fluxes and bounds are normalized relative to the community member's mass fraction, see (4a).

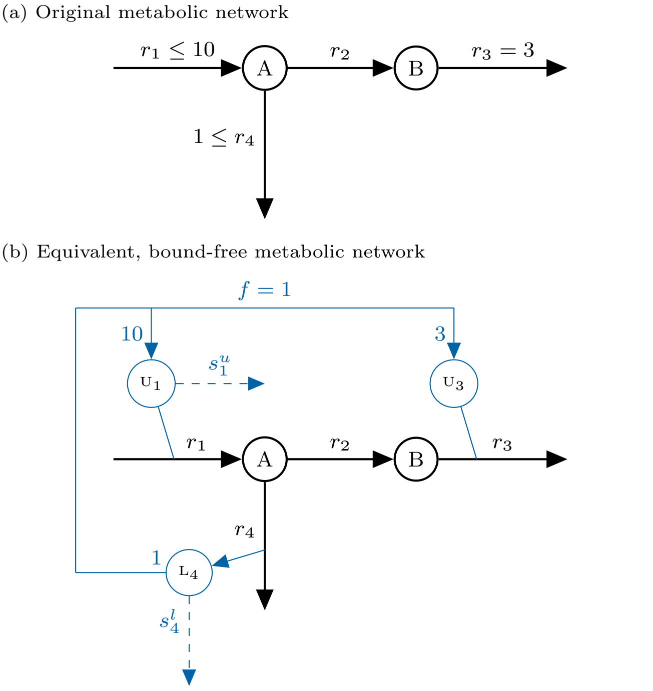


Fig. 1: Reconfiguring a constrained metabolic network (panel a) into an equivalent, bound-free metabolic network (panel b) via the set of Equations (2). Each non-standard bound (in panel a) is substituted with a dummy metabolite (blue circles in panel b). These dummy metabolites are either consumed (for upper bounds) or produced (for lower bounds) by their corresponding reactions. An additional reaction, labeled $f$ (blue line), is introduced to balance these dummy metabolites. It both produces (for upper bounds) and consumes (for lower bounds) these dummy metabolites, with stoichiometric coefficients matched to the corresponding flux bound values. Finally, sink reactions (blue dashed arrows) are added to all dummy metabolites that do not correspond to equality constraints.

Example 1 *Consider the simple constrained metabolic network depicted in Fig. 1a, composed of four irreversible reactions. Flux variability analysis (Mahadevan and Schilling, 2003) reveals the feasible ranges for reaction rates:* $r_1$ *spans* $(4$ *to* $10)\,\mathrm{mmol\,g^{-1}\,h^{-1}}$, $r_2$ *and* $r_3$ *are both* $3\,\mathrm{mmol\,g^{-1}\,h^{-1}}$, *and* $r_4$ *ranges from* $(1$ *to* $7)\,\mathrm{mmol\,g^{-1}\,h^{-1}}$.

*The transformation of the toy network shown in Fig. 1a into its equivalent bound-free network using* (2) *yields the network depicted in Fig. 1b. Upon comparing Fig. 1a and Fig. 1b, it becomes apparent that the flux bounds in Fig. 1a translate into stoichiometric coefficients for newly introduced dummy metabolites, which are either produced or consumed by the new reaction* $f$. *These dummy metabolites function as co-factors, and their availability constrains the respective original reactions* $r_1$ *to* $r_4$. *Any excess can be removed through additional sink reactions, corresponding to the variables* $\boldsymbol{s}^l$ *and* $\boldsymbol{s}^u$. *Note the simplification for the equality constraint* $r_3 = 3\,\mathrm{mmol\,g^{-1}\,h^{-1}}$*:* $U_3$ *is fully consumed by* $r_3$, *precisely determining the flux of* $r_3$ *and rendering both a sink for* $U_3$ *and a dummy metabolite for the lower bound unnecessary. For example, according to* (3) *reaction* $f = 1\,\mathrm{mmol\,g^{-1}\,h^{-1}}$, *which produces* $3\,\mathrm{mmol\,g^{-1}\,h^{-1}}$ $U_3$. $U_3$ *can only be consumed by* $r_3$ *thus enforcing a flux of* $3\,\mathrm{mmol\,g^{-1}\,h^{-1}}$ *trough reaction* $r_3$. *Consequently also* $r_2 = 3\,\mathrm{mmol\,g^{-1}\,h^{-1}}$. $f$ *also produces* $10\,\mathrm{mmol\,g^{-1}\,h^{-1}}$ $U_1$, *which can be consumed by* $r_1$ *or* $s_1^u$, *effectively imposing an upper limit of* $10\,\mathrm{mmol\,g^{-1}\,h^{-1}}$ *on* $r_1$. *Similarly,* $f$ *drains* $1\,\mathrm{mmol\,g^{-1}\,h^{-1}}$ $L_4$, *which enforces a minimum flux of* $1\,\mathrm{mmol\,g^{-1}\,h^{-1}}$ *through* $r_4$. *Any surplus of* $L_4$ *is channeled through reaction* $s_4^l$. *We find a minimum flux of* $r_1 = 4\,\mathrm{mmol\,g^{-1}\,h^{-1}}$ *and a maximum of* $r_4 = 7\,\mathrm{mmol\,g^{-1}\,h^{-1}}$ *upon balancing these fluxes at metabolite A, ultimately fully recovering the limits obtained through flux variability analysis in the original network, as presented above.*

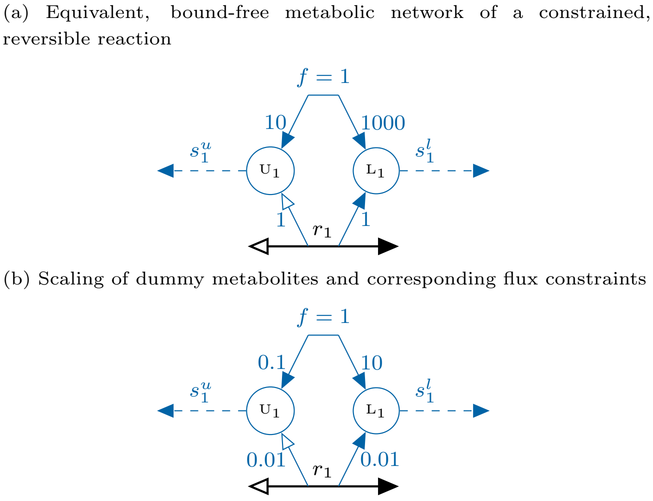


Fig. 2: Equivalent, bound-free metabolic network of a constrained, reversible reaction $-1000 \leq r_1 \leq 10$. Full and open arrows indicate forward and reverse directions of $r_1$, dashed reactions represent sink reactions for the dummy metabolites $\mathrm{L}_1$, and $\mathrm{U}_1$. Panel a shows the reaction $r_1$ with a stoichiometry of 1 for the dummy metabolites $U_1$ and $L_1$. Panel b shows the same reaction as implemented in PyCoMo, yet with stoichiometry coefficients for all dummy metabolites rescaled by a factor of 0.01. This scaling ensures reaction bounds equivalent to panel a, however, the maximum flux through the sink reactions is reduced from 1010 in panel a to 10.1 in panel b.

Example 2 *In computational practice, the commonly adopted capacity constraints* $(l_i, u_i)$ *are usually defined as (-1000,1000) for reversible reactions and (0,1000) for irreversible reactions. For instance, consider the reversible reaction* $-1000 \leq r_1 \leq 10$, *and its equivalent, bound-free network illustrated in Fig. 2a. Note that the negative lower flux bound produces the corresponding dummy metabolite* $L_1$ *by the reaction* $f$.

*Let's assume $r_1$ operates at its maximum forward flux. Consequently, $r_1$ produces $10\,\mathrm{mmol\,g^{-1}\,h^{-1}}$ of $L_1$, while reaction $f$ contributes $1000\,\mathrm{mmol\,g^{-1}\,h^{-1}}$ to the production of $L_1$. Thus, $s_1^l$ needs to drain off a total flux of $1010\,\mathrm{mmol\,g^{-1}\,h^{-1}}$. Such a scenario would be problematic if openCOBRA (Heirendt et al., 2019, 2017; Ebrahim et al., 2013; Jamshidi and Palsson, 2010) default constraints were applied to $s_1^l$.*

*To ensure that sink reactions $s_i^l$ and $s_j^u$ never encounter capacity constraints, we recommend employing considerably larger upper bounds for them, such as $10\,000\,\mathrm{mmol\,g^{-1}\,h^{-1}}$.*

*Increasing bounds for sink reactions can lead to sink reaction fluxes that are significantly larger than all other fluxes in the model, potentially causing numerical issues for the solver. PyCoMo addresses this by enabling the user to adjust the stoichiometric coefficients of associated auxiliary metabolites (Sun et al., 2013). For instance, in Figure 2b, the stoichiometric coefficients of the dummy metabolites have been reduced by a factor of 100 in all reactions producing those metabolites. Despite this adjustment, reaction bounds for $r_1$ are retained. The benefit of this scaling is that it avoids sink reaction fluxes higher than normal reaction fluxes, while still ensuring sufficient efflux for the dummy metabolites, avoiding potential numerical issues in the solver. The default scaling factor for dummy metabolite stoichiometry in PyCoMo is set to $10^{-2}$ as in the example above, but can be adjusted by users when generating a community metabolic model. The flux bounds for the sink reactions of dummy metabolites are scaled accordingly by PyCoMo, to ensuring that no capacity constraints are encountered.*

## 2. Linearisation of community metabolic models

Fluxes in metabolic models are normalized by the dry mass of the model in question. Community metabolic models complicate this system, as the reaction constraints of all reactions of each community member need to be normalized by community mass, but also scaled by the mass fraction of the community member. Further, to comply with the steady-state assumption, the growth rate of all community members needs to be equal. The growth rate of each community member is the amount of this community member's newly produced biomass, divided by the existing mass. The concept of *balanced growth* states that a community can only be in steady state if all community members have equal growth rate on average (Koch et al., 2019). Otherwise, community members with higher growth rates will outgrow slower growing members, altering the community composition and thus violating the steady-state assumption. For a model with $n$ community members $m$; $m \in \{1, ..., n\}$, the mass of the community is set as 1 and the mass of each community member $m$ is equal to the mass fraction $f_m$. Each community member $m$ has a biomass reaction $r^m_{biomass}$ and a growth rate $\mu_m$. As such, in the community metabolic model setting the following constraints apply:

$$\sum_{m=1}^{n} f_m = 1, \tag{4a}$$

$$\mu_{com} = \mu_1 = \mu_2 = ... = \mu_n, \tag{4b}$$

$$\mu_m = \frac{r^m_{biomass}}{f_m} \tag{4c}$$

Thus, the steady-state constraint leads to a bilinear problem in community metabolic models, as the relationship between community member fraction, community member mass and growth rate is non-linear (see equation 4c).

### 2.1. Community and community member mass

To ensure efficient calculation, the model can be linearized by fixing either community member fractions or growth rate. Examples of the model structures can be seen in figures 3 - 5. First of all, the sum of abundance fractions of all community members needs to be 1. This is achieved by each community member's fraction reaction producing a fraction metabolite, which can be converted into a common fraction metabolite $f_{com}$. This common fraction metabolite is coupled to a demand reaction of flux 1, thus ensuring that the sum of fraction reaction fluxes equals 1 (see equations (4) and figure 3).

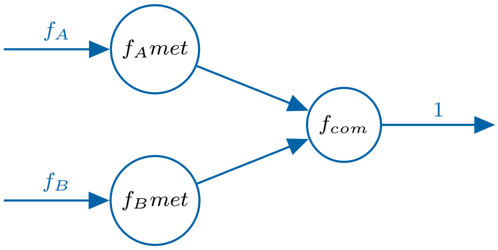


Fig. 3: Use of dummy metabolites to constrain the total mass of the community model. Fraction reactions $f_m$ are introduced for each community member $m$. Fraction reactions have a flux corresponding to the mass fraction of the respective community member and are used to scale the bounds of each member's reactions. The sum of fraction reaction fluxes needs to be equal to the total mass of the community model, which is set to 1. To this end, each fraction reaction produces a dummy metabolite $f_m met$ with a stoichiometric coefficient of 1. Each dummy metabolite $f_m met$ is converted to a community mass dummy metabolite $f_{com}$, connected to a sink reaction of flux 1.

### 2.2. Fixed growth rate

Fixing the community growth rate is achieved by adding an upper bound metabolite to each of the community member's biomass reactions. These upper bound metabolites are not coupled to sink reactions, as they correspond to equality constraints. The production of these biomass bound metabolites is coupled with each community member's fraction reaction. The fraction reaction carries flux equal to the member's mass fraction in the community. The stoichiometric coefficient of the biomass bound metabolites is set equal to the community growth rate (see figure 4). This system leads to the production of biomass metabolites, scaled by the fraction of each member, in the end summing up to the total biomass required for the set growth rate.

$$\mu_{com} = \frac{r^{com}_{biomass}}{\sum_{m=1}^{n} f_m} = r^{com}_{biomass}, \tag{5a}$$

$$r^m_{biomass} = \mu_{com} * f_m, \tag{5b}$$

$$r^{com}_{biomass} = \sum_{m=1}^{n} r^m_{biomass} \tag{5c}$$

This further allows the fractions to be variable, enabling analysis across community compositions.

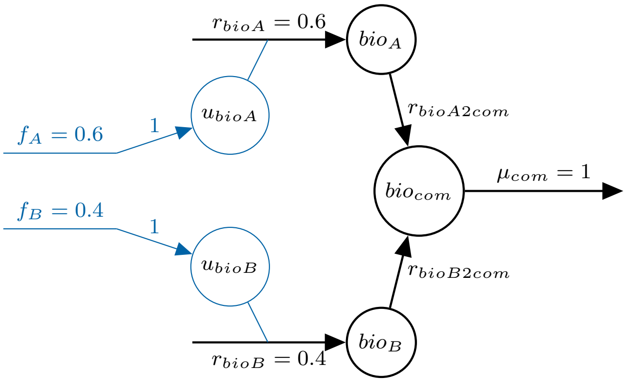


Fig. 4: Community metabolic model structure for fixed growth rate. An example with two community members, A and B, and a fixed community growth rate of 1 is shown. According to equations (5) the flux of the community member's biomass reactions, $r_{bioM}$ is equal to $\mu_{com} * f_m$. In the example shown, solutions for the fraction reaction fluxes are 0.6 for $A$ and 0.4 for $B$. This results in biomass reaction fluxes of 0.6 for $A$ and 0.4 for $B$ as well. The community biomass reaction flux is equal to the community growth rate $\mu_{com}$, as well as the sum of the community members' biomass reaction fluxes.

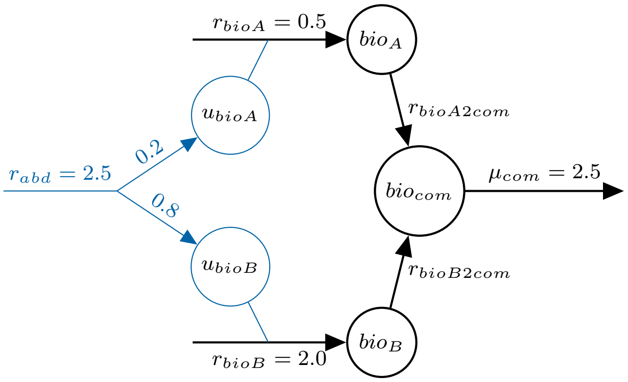


Fig. 5: Community metabolic model structure for fixed community member mass fractions. An example with two community members, A and B, and a fixed abundance profile of $A : 0.2, B : 0.8$ is shown. The stoichiometric coefficients for dummy metabolites in reaction $r_{abd}$ are set to 0.2 for $u_{bioA}$ and 0.8 for $u_{bioB}$. This leads to a balanced biomass production with variable growth rate, while following the constraints of equations (4) and (5). In this example, a community growth rate of 2.5 was chosen as solution. This results in a flux of 2.5 in $r_{abd}$, producing 0.5 of $u_{bioA}$ and 2.0 of $u_{bioB}$. The production of dummy metabolites set the flux of the community biomass reactions accordingly. Ultimately, community biomass is produced in the form of 0.5 of $bio_A$ and 2.0 of $bio_B$, summing up to a community growth rate $\mu_{com}$ of 2.5.

### 2.3. Fixed community member mass fractions

Fixed community member fractions can be achieved by (i) constraining the fraction reactions to the respective fractions and (ii) introducing a reaction ($r_{abd}$) which produces upper bound metabolites for each of the community member's biomass reactions (see figure 5). The flux of reaction $r_{abd}$ is equal to the community growth rate, but is variable in simulation. In $r_{abd}$ the stoichiometric coefficients for the production of the biomass bound metabolites is equal to the fraction of the respective community member, fulfilling the constraints of equations (4) and (5). As in the fixed growth rate, these upper bound metabolites are not coupled to sink reactions, as they correspond to equality constraints.

## 3. Detection of thermodynamically infeasible cycles

Interactions in compartmentalized community metabolic models can create thermodynamically infeasible cycles, that cannot be detected in the member metabolic models alone. To accommodate for this circumstance, a detection of thermodynamically infeasible cycles is included in PyCoMo (accessible via the *get_loops* method of the *CommunityModel* class). The medium of the community metabolic model is set to be empty, allowing no influx of metabolites. Further, the constraints imposed by fixed growth or fixed abundance configurations on the fraction reactions and biomass reactions of the community members are lifted. Then, FVA is used to find reactions that can carry non-zero flux. An example output of the three species biogas community metabolic model (Koch et al., 2019). can be seen in table 1.

In total 31 reactions can carry flux while no medium is present. While all of them are part of thermodynamically infeasible cycles, not all of them are problematic. Many reactions are transport reactions, with a reversible transport being split into two irreversible reactions. As such, they create a cycle, transporting metabolites back and forth indefinitely (e.g. dv_Ac_ex and dv_Ac_up). The same mechanism of reversible reactions being split into two irreversible counterparts can also be found in non-transport reactions, such as the conversion of fumarate to succinate and vice-versa (dv_Fum__Succ_dv_Internal_Species and dv_Succ__Fum_dv_Internal_Species). There is, however, one remaining thermodynamically infeasible cycle with a different pattern, spanning the community members *D. vulgaris* and *M. hungatei*: *D. vulgaris* converts formate into hydrogen and $CO_2$, which are taken up by *M. hungatei* and turned into formate again. Upon detection of such cycles, users are strongly advised to curate the member and/or community metabolic models to resolve these cycles.

## 4. Matching of metabolite namespaces

Matching of boundary metabolites is a key step in allowing members of a community metabolic model to interact. PyCoMo resolves this by either matching via metabolite IDs, or matching via annotation. The latter will treat two boundary metabolites as equal if they have a matching identifier for a database selected by the user in the metabolite annotation. To avoid mapping metabolites to multiple different counterparts, the mapping fails if more than one identifier is present for the selected database in any boundary database. In addition, mass and charge balance is checked for all matched metabolites, and warnings are provided in case of violations.

The success rate of matching metabolite namespaces via annotations is dependent on the extent and similarity of metabolite annotation in the metabolic models about to be merged - not all databases (if any) are provided by all metabolic model generation pipelines, databases may change or provide new identifiers for existing compounds and the protonation state of

| reaction | min_flux | max_flux |
|---|---|---|
| dv_TP_H2_TP_dv_External_Species | -10.0 | 0.0 |
| dv_TP_CO2_TP_dv_External_Species | -10.0 | 0.0 |
| dv_TP_Form_TP_dv_External_Species | 0.0 | 10.0 |
| dv_Fum__Succ_dv_Internal_Species | 0.0 | 10.0 |
| dv_Succ__Fum_dv_Internal_Species | 0.0 | 10.0 |
| dv_Ac_ex | 0.0 | 0.5 |
| dv_CO2_ex | 0.0 | 10.0 |
| dv_Eth_up | 0.0 | 10.0 |
| dv_Form_ex | 0.0 | 10.0 |
| dv_H2_up | 0.0 | 10.0 |
| dv_Ac_up | 0.0 | 0.5 |
| dv_H2_ex | 0.0 | 10.0 |
| dv_Eth_ex | 0.0 | 10.0 |
| dv_Fdred__H2_dv_Internal_Species | -10.0 | 0.0 |
| dv_Fdred__Form_dv_Internal_Species | 0.0 | 10.0 |
| dv_CO2_up | 0.0 | 10.0 |
| mb_CO2_up | 0.0 | 10.0 |
| mb_CO2_ex | 0.0 | 10.0 |
| mh_TP_H2_TP_mh_External_Species | 0.0 | 10.0 |
| mh_TP_CO2_TP_mh_External_Species | 0.0 | 10.0 |
| mh_TP_Form_TP_mh_External_Species | -10.0 | 0.0 |
| mh_Form__CO2_mh_Internal_Species | 0.0 | 10.0 |
| mh_Fum__Succ_mh_Internal_Species | 0.0 | 10.0 |
| mh_Succ__Fum_mh_Internal_Species | 0.0 | 10.0 |
| mh_NADP_Hydrogenase_mh_Internal_Species | 0.0 | 10.0 |
| mh_CO2__Form_mh_Internal_Species | 0.0 | 10.0 |
| mh_CO2_ex | 0.0 | 10.0 |
| mh_CO2_up | 0.0 | 10.0 |
| mh_Form_up | 0.0 | 10.0 |
| mh_H2_ex | 0.0 | 10.0 |
| mh_H2_up | 0.0 | 10.0 |

**Table 1.** Reactions carrying flux while no medium is present. The results shown were generated using a three species biogas community metabolic model (Koch et al., 2019).

compounds can differ between databases, making exact matches impossible.

Thus, the matching of boundary metabolites via annotation provided by PyCoMo is intended for two purposes: (1) If metabolic models were generated by the same model generation pipeline, and annotations are present and unique in a given database for all boundary metabolites, this matching strategy should succeed and allow matching of metabolites regardless of their metabolite ID. (2) When creating a community metabolic model using metabolic models of different metabolic model generation pipelines, where most annotations are present and all are unique in a given database for all boundary metabolites, then matching via annotation can resolve at least the majority of metabolite matching without manual input. As mass and charge balance are checked, all metabolite matches found by PyCoMo should be correct. Users should then resolve all matches that were discovered to be not mass and charge balanced, as well as check all unmatched metabolites for potential missed matches due to any of the reasons provided before.

## 5. Runtime and complexity

The runtime complexity in relation to community size was estimated for three separate tasks: (i) The construction of the community metabolic model, (ii) flux balance analysis (FBA) at equal community member abundance, and (iii) the calculation of all potential exchange metabolites. The runtime was measured on a system running Microsoft Windows 11 with 16GB RAM and an AMD Ryzen 7 5700G using only a single core. To include the effects of diverse input model structures and the complexity of genome-scale metabolic models, the single organism metabolic models were randomly selected from the AGORA (Magnúsdóttir et al., 2017) collection. The three tasks were timed for communities of size 5 to 40 and repeated three times (each with randomly selected members). Figure 6 shows the results of the runtime measurements. The runtime complexity of the community metabolic model construction appears to be roughly linear, while FBA and the calculation of all potential exchange metabolites scale quadratically with the number of community members. It should be noted that fluctuations of an order of magnitude have been observed for the calculation of all potential exchange metabolites. This variability could stem from the differences in community members, but also the implementation of flux variability analysis of COBRApy, used for the calculation in PyCoMo. Further, the

runtime of flux variability analysis (FVA) can be decreased by making use of the parallel implementation in COBRApy. This parallel implementation is not as effective on Windows operating systems as on Linux or MacOS, due to operation system specific implementations of the underlying multiprocessing python library.

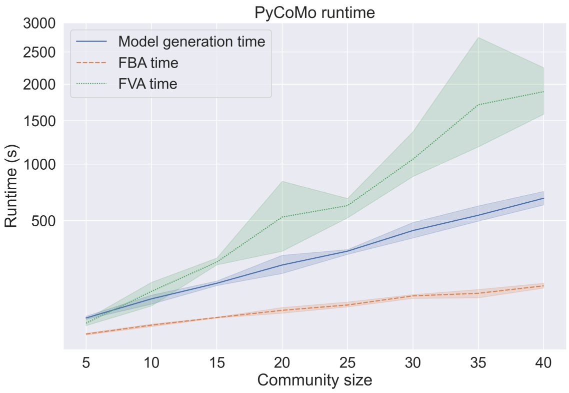


Fig. 6: Runtime measurements for PyCoMo. Single organism metabolic models were randomly sampled from the AGORA collection to generate communities of 5 to 40 members (each in triplicate). The runtime was measured for three tasks of PyCoMo: (i) The construction of the community metabolic model, (ii) flux balance analysis (FBA) at equal community member abundance, and (iii) the calculation of all potential exchange metabolites using flux variability analysis (FVA).

.

## References


A. Ebrahim, J. A. Lerman, B. O. Palsson, and D. R. Hyduke. Cobrapy: constraints-based reconstruction and analysis for python. *BMC systems biology*, 7:1–6, 2013.

L. Heirendt, I. Thiele, and R. M. Fleming. Distributedfba. jl: high-level, high-performance flux balance analysis in julia. *Bioinformatics*, 33(9):1421–1423, 2017.

L. Heirendt, S. Arreckx, T. Pfau, S. N. Mendoza, A. Richelle, A. Heinken, H. S. Haraldsdóttir, J. Wachowiak, S. M. Keating, V. Vlasov, et al. Creation and analysis of biochemical constraint-based models using the cobra toolbox v. 3.0. *Nature protocols*, 14(3):639–702, 2019.

N. Jamshidi and B. Ø. Palsson. Mass action stoichiometric simulation models: incorporating kinetics and regulation into stoichiometric models. *Biophysical journal*, 98(2):175–185, 2010.

S. Klamt, G. Regensburger, M. P. Gerstl, C. Jungreuthmayer, S. Schuster, R. Mahadevan, J. Zanghellini, and S. Müller. From elementary flux modes to elementary flux vectors: Metabolic pathway analysis with arbitrary linear flux constraints. *PLoS computational biology*, 13(4):e1005409, 2017.

S. Koch, F. Kohrs, P. Lahmann, T. Bissinger, S. Wendschuh, D. Benndorf, U. Reichl, and S. Klamt. RedCom: A strategy for reduced metabolic modeling of complex microbial communities and its application for analyzing experimental datasets from anaerobic digestion. *PLOS Computational Biology*, 15(2): e1006759, Feb. 2019. doi: 10.1371/journal.pcbi.1006759.

S. Magnúsdóttir, A. Heinken, L. Kutt, D. A. Ravcheev, E. Bauer, A. Noronha, K. Greenhalgh, C. Jäger, J. Baginska, P. Wilmes, R. M. T. Fleming, and I. Thiele. Generation of genome-scale metabolic reconstructions for 773 members of the human gut microbiota. *Nature Biotechnology*, 35(1):81–89, 2017. doi: 10.1038/nbt.3703. Publisher: Nature Publishing Group.

R. Mahadevan and C. H. Schilling. The effects of alternate optimal solutions in constraint-based genome-scale metabolic models. *Metabolic engineering*, 5(4):264–276, 2003.

Y. Sun, R. M. Fleming, I. Thiele, and M. A. Saunders. Robust flux balance analysis of multiscale biochemical reaction networks. *BMC bioinformatics*, 14(1):1–6, 2013.
